# Transradial Approach Superior to Transfemoral Approach for Guide Catheter Engagement of Right Coronary Artery

**DOI:** 10.4137/ccrep.s733

**Published:** 2008-04-16

**Authors:** Samir B. Pancholy

**Affiliations:** Department of Cardiology, Mercy Hospital, 746, Jefferson Avenue, Scranton, PA.

## Introduction

Percutaneous coronary intervention (PCI) has become the treatment of choice for patients with symptomatic one-vessel coronary artery disease especially when they fail medical therapy alone. Guide catheter support is essential to perform PCI. Transfemoral access is the most commonly used access for PCI. Most guide catheter shapes are designed to allow co-axial engagement from the femoral approach. We have observed that transradial approach frequently provides superior guide support in patients where transfemorally placed guides are either suboptimal or on occasion not successful in engaging the target vessel. We describe a case of PCI of right coronary artery arising from right sinus of valsalva, where engaging the ostium from right femoral access was unsuccessful despite aggressive attempts, with subsequent transradial approach successfully allowing guide-catheter placement with extreme ease, followed by successful completion of ostial right coronary artery stenting.

## Case Details

**Figure 1:**
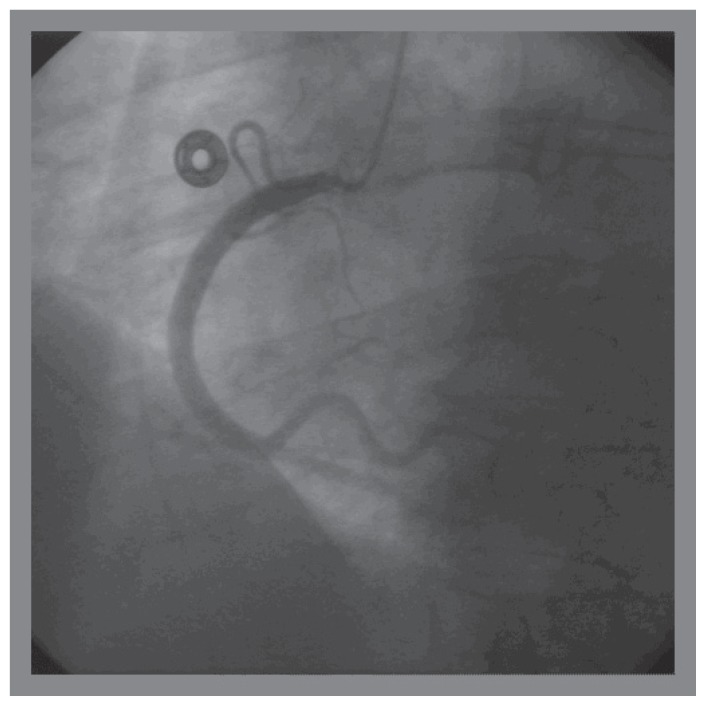
Cannulation of the right coronary artery from right radial access, with 4 french JR4 catheter for diagnostic coronary angiography.

A 72 years old female with history of hypertension, presented with retrosternal and interscapular pain upon minor exertion, for 48 hours. Inferior wall motion abnormality was seen on echocardiogram. EKG and Troponin I was normal. Cardiac catheterization was performed by using 4 french access from right radial artery, using 4 french JL3.5 and JR4 catheters. An 80% ostial stenosis involving the right coronary ostium was noted. No catheter waveform change was noted. 4 french JR4 catheter was placed into the right coronary artery without any difficulty (Fig. 1). LV function was normal. Manual pressure was used to achieve hemostasis.

**Figure 3:**
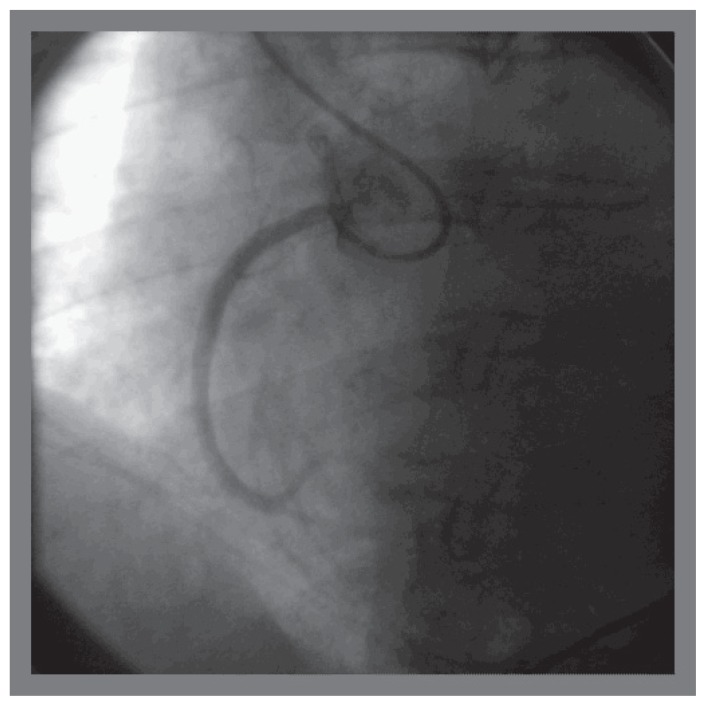
Attempt at selective cannulation of right coronary artery ostium using a MAC 3.0 catheter (unsuccessful), using right femoral access.

**Figure 4:**
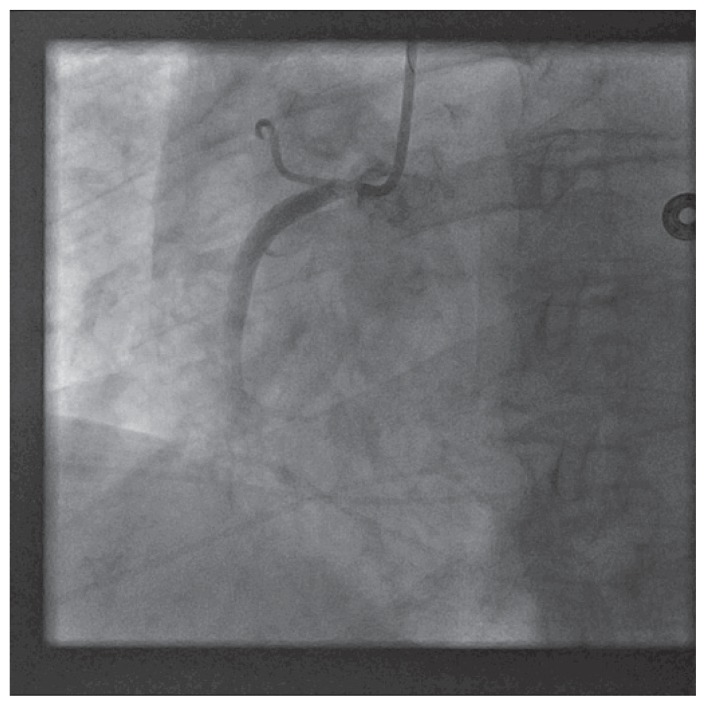
Successful cannulation of right coronary artery ostium using JR4 guide catheter using right radial access (first attempt).

The patient was brought back to the catheterization laboratory 24 hours later for percutaneous coronary intervention, and transfemoral approach was used selected in view of large reference lumen diameter of the right coronary artery and hence anticipated need for large profile access. A 7 french introducer sheath was placed in the right femoral artery in a standard fashion. Attempts were made using JR4, JR3.5, Williams, AL1, LIMA, Hockey-stick, Multipurpose, Champ, Kiez right 1, AR1, MAC 3, MAC 3.5, catheters for approximately two hours, to engage right coronary artery ostium (Figs. 3–4). Altering the guide characteristics with use of the stiff end of 0.035″/260 cm guidewire and subsequent manipulation was also attempted. All of these attempts were unsuccessful. After using 250 ml of non-ionic contrast, the procedure was terminated.

**Figure 5:**
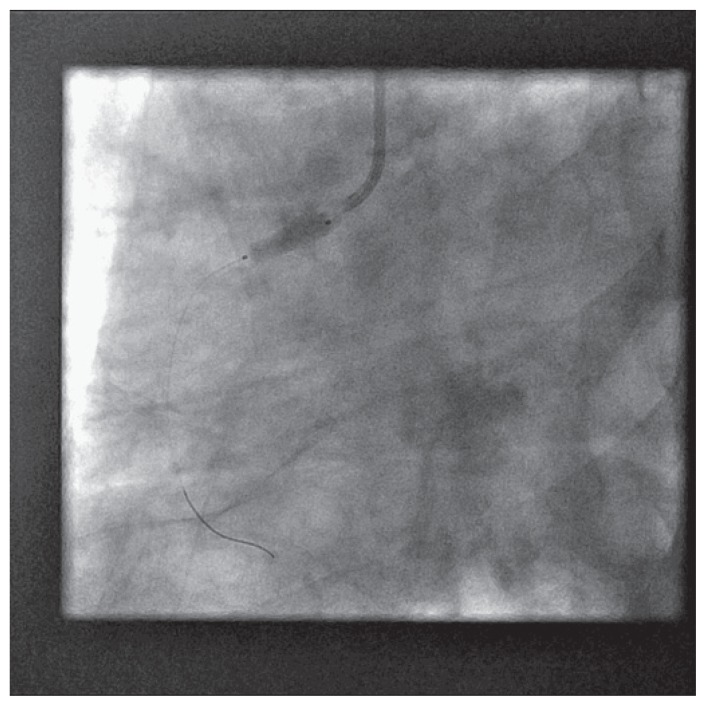
Cutting balloon (4.0/15mm) inflation at right coronary ostium using JR4 guide catheter from right radial access.

**Figure 6:**
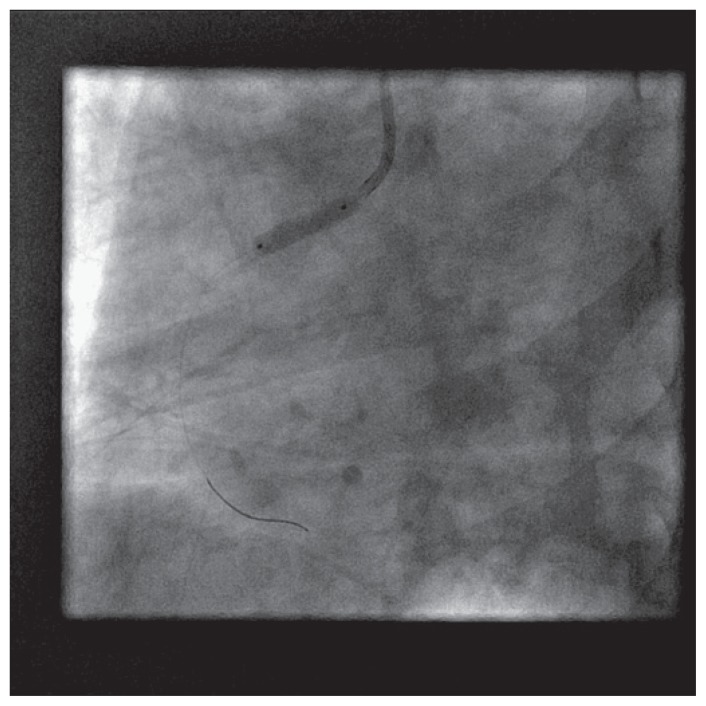
Stent deployment (5.0/18 mm Herculink) at right coronary artery ostium using JR4 guide catheter from right radial access.

**Figure 7:**
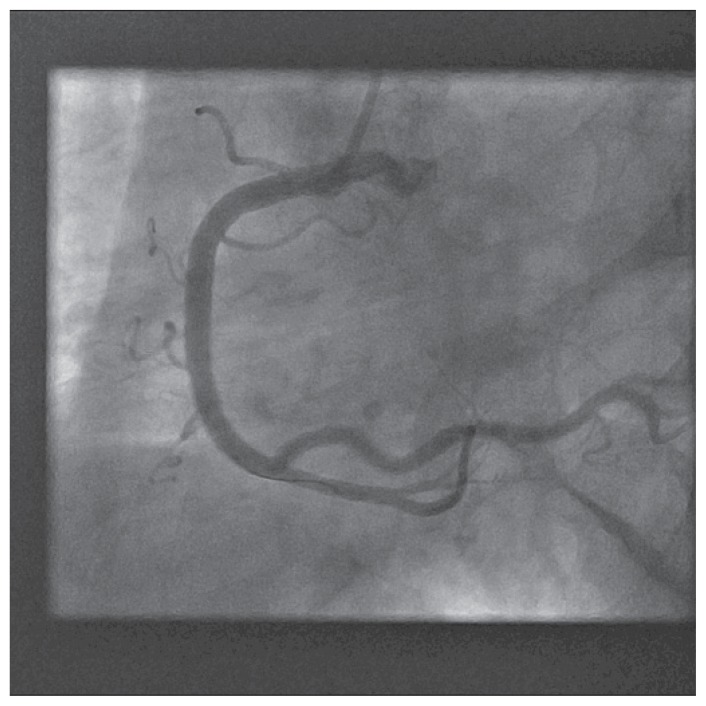
Final result after transradial intervention.

The patient was brought back to the catheterization laboratory for a final attempt at PCI from right radial artery access. 6 french Radiofocus Glidesheath (Terumo Medical) was introduced in the right radial artery and 6 french JR4 guiding catheter was placed in the ascending aorta. Right coronary ostium was engaged at the first attempt (Fig. 5). Catheter waveform ventricularization was noted. The catheter was withdrawn promptly. A 0.014′/190 cm Hi-Torque floppy guidewire was placed in the catheter and the right coronary ostium was promptly re-engaged at first attempt. The guidewire was promptly placed in the distal right coronary artery and 4.0/15 mm Cutting balloon was used to pre-dilate the right coronary ostium with 1 inflation at 14 atmospheres for 120 seconds (Fig. 6). A 5.0/18 mm Herculink stent was deployed at the right coronary ostium at 16 atmospheres for 20 seconds. The stent was post-dilated with the same balloon with two inflations at 20 atmospheres for 15 seconds each (Fig. 7). Excellent angiographic result was obtained, confirmed in two orthogonal views.

## Discussion

Despite major advances in PCI, basic necessities such as co-axial and strong guide-catheter support remain the foundation for success. Transfemoral access still remains the most common access for PCI in the U.S. Multiple catheter shapes have been developed to accommodate anatomic variations, but on occasion none of these catheters allow for one to engage the target vessel. Multiple factors play a role in making the anatomy insurmountable. Anomalous aorto-ostial anatomy is the most common scenario for difficult target vessel access.^1^–^4^ Choosing a suitable guide catheter based on location of anomalous origin and aortic root size, frequently allows the operator to access these anomalous or variant ostia.^5^,^6^ Certain dedicated guide catheter shapes are also available for this purpose.^7^ Catheter manipulation with specific maneuvers has been successful in certain situations.^8^ Right radial approach has been described to be successful in accessing anomalous left sinus of valsalva origin right coronary artery.^9^

Infrequently, the unfolding of the ascending aorta, aortic arch as well as descending thoracic aorta and iliac vessels, prohibits the catheter engagement of a non-anomalous coronary ostium. We have encountered this problem more frequently using transfemoral approach, and have noticed a surprising lack of these situations using transradial approach. One reason for this difference is the absence of the influence of aorto-iliac anatomy on the behavior of the coronary guiding catheter in the ascending aorta. By traversing a shorter segment of aorta, using TRI, the effects of the distal anatomy on proximal catheter behavior are decreased. This is especially noticeable in right coronary interventions. The right coronary catheters when placed from right radial access, provide superior back-up support, as opposed to the support provided by the same catheter from femoral approach, in view of the fact that transradial placement allows for these catheters to obtain added support from the contralateral aortic wall, similar to the behavior of the extra-backup series guide catheters for the left coronary artery.

As PCI is now performed on a wider variety of patients with more elderly in the population undergoing PCI, encountering these procedural situations has become more frequent. TRI from right radial access offers a completely different geometric approach to the ascending aorta, and hence provides an option that femoral approach or catheter shape variation, does not allow for. TRI from the left radial artery mimics the transfemoral geometry. Hence for guide-catheter placement one could argue that transradial approach is certainly offering the benefits of transfemoral approach if left radial artery is used and a different orientation to the aorto-ostial junction if the right radial artery is used. We recommend switching to transradial approach when femoral approach does not succeed in engaging the target coronary artery. In order to successfully offer PCI to all subsets of patients, the interventionalist will need to be able to perform transradial intervention.

## Figures and Tables

**Figure 2:**
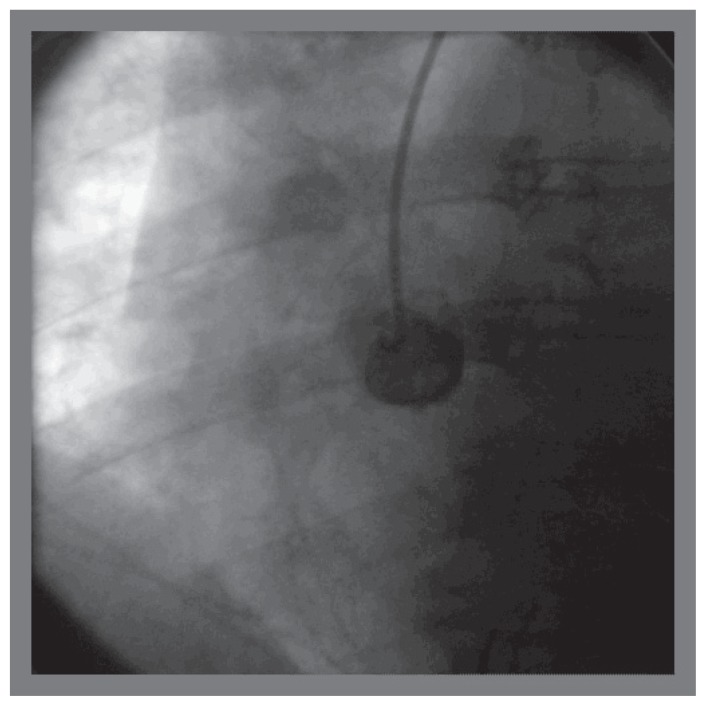
Attempt at selective cannulation of right coronary artery ostium using JR4 guide catheter (unsuccessful), using right femoral access.
